# Influenza associated excess mortality in Germany, 1985 – 2001

**DOI:** 10.1186/1742-7622-2-6

**Published:** 2005-06-21

**Authors:** Phillip Zucs, Udo Buchholz, Walter Haas, Helmut Uphoff

**Affiliations:** 1 Swiss Federal Office of Public Health, Berne, Switzerland; 2 Robert Koch-Institut, Berlin, Germany; 3 Staatliches Untersuchungsamt Hessen, Dillenburg, Germany

## Abstract

Influenza-associated excess mortality is widely used to assess the severity of influenza epidemics. In Germany, however, it is not yet established as a routine component of influenza surveillance. We therefore applied a simple method based on the annual distribution of monthly relative mortality (relative mortality distribution method, RMDM) to a time-series of German monthly all-cause mortality data from 1985–2001 to estimate influenza-associated excess mortality. Results were compared to those obtained by cyclical regression.

Both methods distinguished stronger from milder influenza seasons, but RMDM gave the better fit (R^2 ^= 0.80). For the years after reunification, i.e. 1990/91 through 2000/01, RMDM yielded an average of 6900 (conservative estimate) to13600 influenza-asssociated excess deaths per season (crude estimate). The most severe epidemics occurred during subtype A/H3N2 seasons. While German all-cause mortality declined over the study period, the number of excess deaths displayed an upward trend, coinciding with an increase of the proportion of the elderly population.

## Introduction

In the Northern hemisphere, influenza is a seasonal disease with high epidemic potential. Excess mortality due to influenza is frequently used as one important parameter to assess the severity of epidemics [1].

In non-pandemic years, influenza-associated death is mainly restricted to the elderly and people with underlying chronic illnesses [2,3]. However, analyses of death certificates show that clinicians often do not attribute influenza-related deaths to influenza, but rather to a pre-existing underlying condition. Influenza-associated deaths may therefore be hidden not only among cases of pneumonia but among other causes of death such as cardiovascular events or metabolic disorders [4]. Hence, all-cause mortality has been found to be more complete and accurate for assessing the total impact of influenza on mortality [1].

Conceptually, influenza-associated excess mortality is estimated as the difference between observed mortality during the influenza season and baseline values to be expected during that time span if influenza were absent. Different approaches to obtain estimates of the baseline have included cyclical regression [5,6], Poisson regression [4], multiple linear regression [7,8] and autoregressive integrated moving average (ARIMA) models [9].

In Germany, an influenza sentinel surveillance system was established in August 1992 (Arbeitsgemeinschaft Influenza: AGI). It consists of a nationwide network of private practice physicians who report data on acute respiratory infections and send throat swabs from a sample of patients to the Influenza Reference Laboratory for virus isolation and typing [10]. Before August 1992, German influenza surveillance relied exclusively on reference laboratory results, was based on fewer samples and included a considerable proportion of hospital patients in addition to patients seen by private physicians [11]. The current system uses excess incidence of physician consultations and hospitalisations due to acute respiratory infections to assess the severity of influenza seasons. Mortality data have not been analysed routinely, partly because German vital statistics become available with at least six months delay. As a step towards the incorporation of mortality data into monitoring the impact of influenza in Germany, we applied two types of models to a time-series of German all-cause mortality data from January 1985 through December 2001: cyclical regression and a method based on the distribution of monthly relative mortality (relative mortality distribution model, RMDM). Besides estimating excess mortality linked to influenza in Germany, our goal was to evaluate the two approaches in terms of simplicity, practicality and goodness-of-fit.

## Methods

### Data source and software

We obtained all-cause mortality data by month of report from 1985 to 2001 and population data for the same time period from the German National Bureau of Statistics. Weekly data or age-stratified monthly data were not available. We adjusted for the variable length of months by dividing each value by that month's number of days, multiplying by 365 and dividing by 12. For the period January 1985 through December 1990, we used only West German mortality data in the analyses because East German data were incomplete. For the period from January 1991 onwards, i.e. after reunification in October 1990, we used data from the entire country. Virologic surveillance data from the German Influenza Reference Laboratory served to identify months with influenza activity between January 1985 and August 1992. After August 1992, morbidity data from the then founded sentinel influenza surveillance system were additionally available.

All analyses were done using Microsoft Excel 97 (Redmond, WA, USA).

### Cyclical regression

We used a "Fast Fourier Transform" to identify significant cyclical components in our time-series. As this procedure requires powers of 2 for the number of data-points, we looked at two overlapping time periods of 128 months each from January 1985 through August 1995 and from January 1991 through August 2001. In each of the two time-series, stationarity had to be achieved for any further modelling. Therefore the variance was stabilized by log transformation and the linear trend removed by linear regression and subtraction. We then used a least squares approach to fit a cosine curve described by the equation

_t _= a + bt + ∑Rcos(ωt + θ)

where  is the expected all-cause mortality, a and b are intercept and slope, respectively, of a linear term, t is the index for month of reported death, R is the amplitude of the periodic variation, ω is its frequency and θ is the phase.

From this model, we omitted any month for which surveillance had indicated influenza activity and during which observed mortality exceeded modelled mortality. Refitting of the model yielded baseline all-cause mortality values which would be expected in the absence of influenza activity. 90% CI were calculated based on the standard deviation of the residuals according to the formula



where _t _is the expected all-cause mortality in a given month t, y_O_is any mortality observed during the study period, y_E _is any mortality expected in the same month as y_O_, n is the number of observations and dof is the number of degrees of freedom.

Data were finally restored to their original state by adding the linear trend that had initially been subtracted, followed by exponential transformation. The fit of the model with baseline mortality was assessed by the coefficient of determination, R^2^.

### Relative mortality distribution model

**Figure 1:**
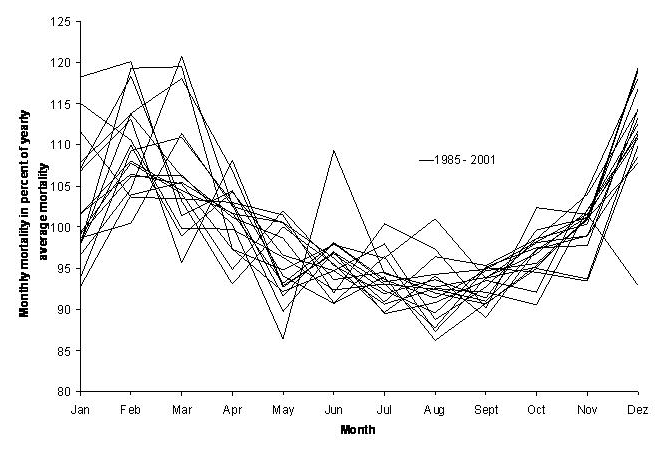
Annual distribution of monthly all-cause mortality relative to the yearly average mortality, West Germany (1985 – 1990) and unified Germany (1991 – 2001).

The time period under study was January 1985 through August 2001. We observed that during this period the ratios of each month's mortality and that year's average mortality (= relative mortality) followed a fairly constant pattern (Figure 1). We calculated the mean relative mortality of all corresponding months across the years studied. Expected mortality for any given month could then be modelled by multiplying the respective mean with the total mortality observed in that year. Months in which influenza activity was reported and in which observed mortality exceeded the model values were omitted and mean monthly percentages recalculated by minimising the residuals for the remaining months. This step was repeated a second time resulting in the final model of baseline mortality. Upper and lower 90% confidence intervals (CI) were calculated based on the standard deviation of the residuals as described for cyclical regression. Again, the fit of the model with baseline mortality was assessed by the coefficient of determination, R^2^.

### Excess mortality computation and further analyses

The principle was the same for both approaches. Influenza-associated excess mortality for each season was estimated as the sum of all positive monthly excess values (i.e. observed minus expected mortality after refitting of the model) within the period of increased influenza activity as indicated by surveillance data. In addition to this crude estimate, a more conservative estimate was obtained by subtracting the upper 90% confidence limits instead of the model point estimates from the observed mortality values.

We compared estimates by influenza season, estimation approach and influenza type/subtype. Type/subtype-specific excess mortality was assumed to correspond to a type/subtype's proportion among human isolates within a given season. Type/subtype-specific average seasonal excess mortality was estimated based on seasons in which a type/subtype predominated, i.e. accounted for more than 50% of the seasonal total of human isolates.

The number of influenza-associated excess deaths was estimated using the RMD model that was developed for the entire time span (1985–2001). However, as reunification enlarged the study population by about 25%, calculations were restricted to unified Germany from January 1991 until August 2001.

## Results

Between January 1985 and August 2001, seasonal influenza activity had occurred in Germany almost annually during one or more months between December and April. The adjusted monthly all-cause mortality ranged between 76 and 113 per 100,000 population and followed a seasonal pattern peaking in winter months.

**Figure 2:**
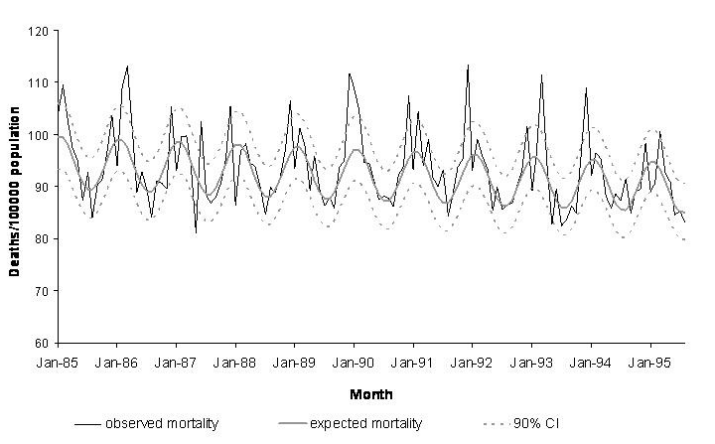
Observed mortality and mortality to be expected in the absence of influenza, as estimated by cyclical regression, West Germany (1985 – 1990) and unified Germany (1991 – 2001).

Fourier analysis of observed mortality indicated one cyclical component of 12 months length for both the period January 1985 through August 1995 and the period January 1991 through August 2001. R^2 ^values obtained by cyclical regression were 0.71 and 0.73 for the first and second study period, respectively. The resulting models led to average seasonal excess mortality estimates of 16.1 and 17.4 deaths per 100,000 population for the first and second study period, respectively. Seasonal estimates ranged between 2.2 and 44.2 excess deaths per 100,000 population (Table 1). Conservative estimates averaged at 6.8 and 7.7 deaths per 100,000 population and season for the first and second study period, respectively (range: 0 to 25.8). The difference between the crude and conservative estimate was 10.3 per 100,000 population (61% of the crude estimate) for the period 1985–1995 and 12.0 per 100,000 population (62% of the crude estimate) for the period 1991–2001. Figure 2 shows a graphic representation of cyclical regression applied to the period 1985–1995.

**Table 1:** Influenza-associated excess mortality per 100,000 population in West Germany (1984/85–1989/90) and united Germany (1990/91–2000/01), by method of estimation

	Cyclical Regression		Relative Mortality Distribution	
		
Influenza season	Crude estimate^a^	conservative^b^	Crude estimate^a^	conservative^b^
1984 / 85^c^	17.9	3.2	22.0	8.7
1985 / 86^c^	25.4	12.9	23.4	13.9
1986 / 87^c^	9.3	1.8	2.9	0.0
1987 / 88^c^	2.2	0.0	3.5	0.0
1988 / 89^c^	10.3	3.9	5.9	0.0
1989 / 90^c^	35.7	17.0	30.8	16.3
1990 / 91	7.4 / 5.9 ^d^	1.5 / 0 ^d^	4.6	0.1
1991 / 92	21.8 / 19.6 ^d^	12.6 / 11.1 ^d^	15.8	4.2
1992 / 93	21.2 / 21.4 ^d^	11.2 / 11.2 ^d^	18.9	12.3
1993 / 94	16.5 / 16.6 ^d^	9.0 / 8.8 ^d^	7.8	2.8
1994 / 95	9.0 / 11.7 ^d^	1.2 / 2.4 ^d^	9.7	3.4
1995 / 96	44.2	25.8	40.6	26.1
1996 7 97	8.0	1.0	15.0	5.5
1997 / 98	10.9	1.2	11.3	2.6
1998 / 99	26.9	15.4	26.7	19.3
1999 / 2000	12.9	5.5	19.3	9.6
2000 / 01	12.7	2.0	13.5	7.0

1984/85–1994/95	176.7	74.3	145.2	61.6
1990/91–2000/01	190.9	84.4	183.2	92.9
1984/85–2000/01	292.3 / 291.6	125.2 / 123.2	271.7	131.8

^a ^observed minus expected mortality

^b ^observed minus upper 90%confidence limit of expected mortality

^c ^West German excess mortality only

^d ^For cyclical regression two models were built, one for the time period 1984/85–1994/95 and one for 1990/91–2000/01, resulting in two estimates for the seasons 1990/91–1994/95

**Figure 3:**
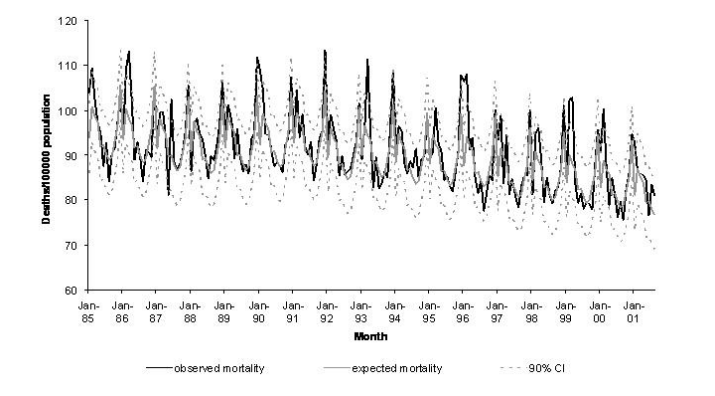
Observed mortality and mortality to be expected in the absence of influenza, as estimated by a the relative mortality distribution approach, West Germany (1985 – 1990) and unified Germany (1991 – 2001).

Applying RMDM to the period 1985–2001 gave an R^2 ^value of 0.80 for the total period. If calculated separately for the two periods as studied by cyclical regression, R^2 ^was 0.72 for the first and 0.80 for the second period. Estimated average seasonal influenza-associated excess mortality amounted to 16.0 (range: 2.9 to 40.6) excess deaths per 100,000 population. The conservative estimate was 7.8 (range: 0 to 26.1) deaths per 100,000 population (table 1). The difference between the two estimates was 8.2 per 100,000 population (51% of the crude estimate). Figure 3 displays the modelled influenza-free mortality and the observed mortality from 1985–2001.

In most seasons, especially those with high excess mortality, the methods yielded similar crude estimates. Comparing total excess mortality over the two 10-year periods that were required for cyclical regression, RMDM crude estimates were lower (145.2 vs. 176.7 and 183.2 vs. 190.9). For the period 1985–1995 RMDM also yielded a lower conservative estimate (61.6 vs. 74.3) while for the second period it was higher (92.9 vs. 84.4) than the one obtained by cyclical regression.

In 9 of the 17 influenza seasons, Influenza A/H3N2 accounted for more than 50% of human isolates at the National Reference Laboratory and caused an estimated average mortality of 15.4 per 100,000 population in those seasons (Table 2). Three seasons were dominated by Influenza A/H1N1 (average mortality 6.7 per 100,000) and 5 seasons by Influenza B (average mortality 11.8 per 100,000).

**Table 2:** Influenza-associated excess deaths per 100,000 population in West Germany (1984/85–1989/90) and united Germany (1990/91–2000/01), estimated by relative mortality distribution modelling (crude approach) and stratified by influenza type/subtype. Excess mortality figures are only displayed for the seasonally predominating type/subtype (>50% of human isolates).

		A/H3N2		A/H1N1		B	
				
Influenza season	Total EM	% of isolates	EM	% of isolates	EM	% of isolates	EM
1984 / 85	22.0	97	21.4	1		2	
1985 / 86	23.4	1		0		99	23.1
1986 / 87	2.9	0		97	2.8	3	
1987 / 88	3.5	100	3.5	0		0	
1988 / 89	5.9	19		76	4.5	4	
1989 / 90	30.8	82	25.3	0		18	
1990 / 91	4.6	0		15		85	3.9
1991 / 92	15.8	69	10.8	31		0	
1992 / 93	18.9	16		0		84	15.8
1993 / 94	7.8	100	7.8	0		0	
1994 / 95	9.7	19		2		79	7.7
1995 / 96	40.6	55	22.4	42		3	
1996 7 97	15.0	39		6		55	8.2
1997 / 98	11.3	96	10.5	7		1	
1998 / 99	26.7	67	17.9	0		33	
1999 / 2000	19.3	99	19.0	1		0	
2000 / 01	13.5	0		95	12.9	5	
Average^a^			15.4		6.7		11.8

^a ^Excess mortality

The overall number of excess deaths for the period 1990/91–2001 from RMDM (Table 3) was between 3670 and 33234 influenza-associated excess deaths for the crude estimate and between 60 and 21365 for the conservative estimate. The average number of excess deaths for the 11 seasons was estimated to lie between 6906 (conservative estimate) and 13601 (crude estimate).

**Figure 4:**
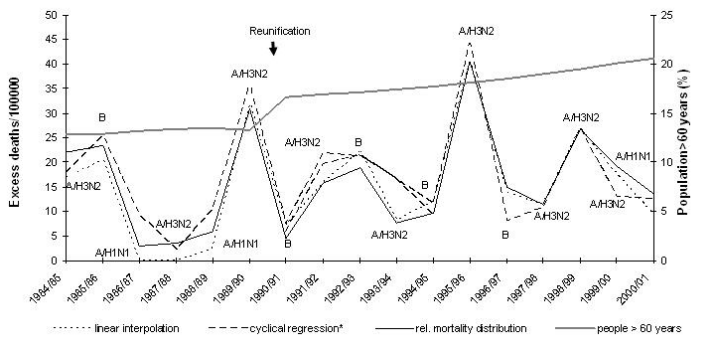
Dominant influenza strains (>50% of isolates), influenza-associated excess deaths per 100000 population and season, by estimation method, and proportion of population over 60 years, West Germany (1985 – 1990) and unified Germany (1991 – 2001).

Between 1984/85 and 2000/01 excess mortality peaked roughly every four to five years and showed a rising trend in parallel with the proportion of the population over 60 years of age (Figure 4). The two largest peaks were due to influenza A/H3N2, and were detected by both approaches including the conservative estimates.

**Table 3:** Influenza-associated excess deaths in Germany, 1991–2001, estimated by relative mortality distribution modelling

Influenza season	Crude estimate^a^	Conservative^b^
1990 / 91	3670	60
1991 / 92	12676	3345
1992 / 93	15281	10003
1993 / 94	6304	2241
1994 / 95	7911	2738
1995 / 96	33234	21365
1996 / 97	12338	4535
1997 / 98	9296	2172
1998 / 99	21907	15854
1999 / 00	15854	7896
2000 / 01	11136	5757

Total	138471	70209

Average	13601	6906

^a ^observed number of deaths minus expected number of deaths

^b ^observed number of deaths minus upper 90% confidence limit of expected number of deaths

## Discussion

This paper presents a systematic account of recent influenza-associated excess mortality in Germany. For the decade following reunification, the average seasonal number of excess deaths due to influenza ranged between 6906 (conservative estimate) and 13601 (crude estimate), with the most severe epidemics associated with the A/H3N2 subtype.

While German all-cause mortality declined over the study period, excess deaths appeared to have been rising. This increase seemingly contradicts the growing number of sold doses of influenza vaccine in Germany [12]. However, during the same time period the proportion of the elderly in the German population has also increased and may be the underlying factor that is driving excess deaths. A similar explanation for equally paradox trends was suggested in a recent analysis of US mortality data [13].

The two methods were able to differentiate severe influenza seasons from mild ones detecting excess mortality peaks in 1989/90 and 1995/96 which were also the influenza seasons with the highest morbidity during the study period.

Cyclical regression is an established technique for time-series analysis and has been one of the methods used by the U.S. Centers for Disease Control and Prevention to estimate influenza-associated excess mortality [6]. It requires an understanding of trigonometrics and may not be straightforward to apply for non-epidemiologists or non-statisticians.

The modelling approach that is based on the annually recurring pattern of relative mortality distribution is simple, practical and yields excess mortality estimates in line with those obtained by cyclical regression. Unlike Fourier cyclical regression, however, it neither requires a certain number of time units nor was it necessary to assume linear trends in annual mortality. Because variations of year-to-year estimates may not be linear, the model relies only on the average annual mortality and is therefore more flexible than the cyclical regression model.

The methods yielded crude excess estimates that were not very far apart. As the exact extent of influenza-associated excess mortality is unknown, the accuracy of the estimations could not serve to evaluate the approaches. The goodness-of-fit of RMDM was slightly superior and the approach seemed better than cyclical regression at capturing irregularities of the mortality curve every December and January, that are probably due to holiday season-associated delays in death registration by time of report (Fig. 2 and 3). Although the better fit may be explained by the larger number of parameters included in the model, the easier handling and higher flexibility made us favour RMDM.

The influenza-associated seasonal excess mortality of 16 per 100,000 population that we obtained for Germany using the RMDM was plausible when compared with previously published numbers from other countries. Researchers in the Netherlands, the USA and Switzerland estimated for their countries an influenza-related annual mortality of 14, 19.6 and 21.6 per 100,000 population, respectively [4,13,14].

So far, mortality data have not been incorporated in the routine surveillance of influenza in Germany since they become available only after a lag of up to one year. However, mortality data are not only necessary to calculate the burden of influenza for a given season but may also serve as an efficient surveillance tool [15] in particular for pandemic situations. We found that the more severe influenza seasons in Germany were associated with predominance of A/H3N2 followed by B and A/H1N1. This general trend is in line with the findings of Thompson in the USA [13]. The weak effects of A/H1N1 seasons have probably to do with the fact that this subtype has been circulating continuously since 1918 except for an interval between 1957 and 1977. It had thus ample time to adapt to the human population and vice versa.

The study has the following limitations. As influenza seasons usually last six to ten weeks with a peak phase of one to three weeks, we would have ideally preferred to use weekly mortality data. However, only monthly mortality data were available from the German National Bureau of Statistics. The use of weekly data might have increased model resolution and thus estimate precision.

To minimize the likelihood of taking deaths into account which were rather due to winter-related causes other than influenza, we attributed excess mortality to influenza only if it occurred during months with influenza activity as indicated by sentinel and virologic surveillance data. However, an important alternative cause of death during influenza seasons is respiratory syncytial virus (RSV) infection [13,16]. The RSV seasons from 1994/95 to 1996/97 started late (December to January) and ended between March and May. From 1997/98 to 2001, it seems that late and early seasons (start in September to October) have been alternating [17]. These data indicate that overlap between RSV and influenza seasons and thus overestimation of influenza-associated excess mortality cannot be ruled out

We fitted the initial models to the entire time-series including epidemic months. This might have led to some overestimation of expected and underestimation of influenza-associated excess mortality, but this method has been used previously [1,18]. Furthermore, although we tried to model expected mortality by removing all months with excess mortality, we may have falsely retained some months in which influenza-associated mortality was present, thereby again overestimating expected and underestimating excess mortality. On the other hand, two factors may have caused some overestimation of influenza-associated excess mortality. First, the use of all-cause mortality is likely to take deaths into account that are not related to influenza [13]. Second, the approach of ignoring negative "excess" may entail contributions to excess mortality from positive random deviations that would have otherwise been cancelled out by negative random deviations.

Finally, our estimates are across all age-groups as age-specific mortality data were not available. Age-specific data would not only improve the quality of the models and the estimates, but would also allow to target public health messages more precisely. For the future, timely, weekly and age-specific mortality data are highly desirable to build better models, calculate burden of disease more precisely and allow integration into routine influenza surveillance.

## Authors' contributions

PZ and UB carried out cyclical regression and drafted the manuscript. HU developed and applied the relative mortality distribution model and drafted the manuscript. WH revised the manuscript.

## Note

*Cyclical regression was separately applied to the periods 1984/85 – 94/95 and 1990/01 – 2000/01. Both curves are displayed in the figure, resulting in an overlap during the years 1990/91–1994/95
